# Experimental Investigation of High Temperature-Resistant Inductive Sensor for Blade Tip Clearance Measurement

**DOI:** 10.3390/s19010061

**Published:** 2018-12-24

**Authors:** Ziyu Zhao, Zhenxia Liu, Yaguo Lyu, Yajun Gao

**Affiliations:** School of Power and Energy, Northwestern Polytechnical University, Youyi West Road 127#, Xi’an 710054, China; zziyu@mail.nwpu.edu.cn (Z.Z.); zxliu@nwpu.edu.cn (Z.L.); 1620457036@mail.nwpu.edu.cn (Y.G.)

**Keywords:** inductive sensor, tip clearance, turbine blade, temperature influence

## Abstract

Turbine tip clearance of aero-engine is important to engine performance. Proper control of rotor tip clearance contributes to engine efficiency improvement and fuel consumption reduction. Therefore, accurate tip clearance measurement is essential. The inductive measurement method is one of the non-contact distance measurement methods, which has the characteristics of high sensitivity, fast response speed and strong anti-interference ability. Based on the principle of inductive sensor measuring tip clearance, the ambient temperature change will cause the material electromagnetic performance change for the conductivity and permeability varies with temperature. The calibration experiment was conducted to obtain the sensor resolution and sensing range. The effect of temperature on sensor parameters was extracted from high temperature experiment data. Results show the resolution of planar coil made of platinum wire can be 10 μm and the maximum sensing range can reach 5 mm. At temperature from 500 ℃ to 1100 ℃, coil inductance almost does not change with temperature while coil resistance varies exponentially with temperature, that means the coil inductance variation can reflect the tip clearance change and resistance can indicate the measuring temperature.

## 1. Introduction 

The blade tip clearance of gas turbine is significant for its performance and efficiency. Therefore, precise measurement of tip clearance is the premise of accurate design and optimization the tip clearance [1,2,3]. The study of sensor with high precision and high resolution for tip clearance is necessary and crucial. Numerous non-contact measurement technologies are developed, including microwave, optical, capacitive, and inductive.

Mark R.W. et al. [4,5,6] from NASA Glenn Research Center started effort on microwave method applying to tip clearance measurement since 2003. The microwave sensor probe is able to operate at extremely high temperature and is unaffected by contaminants in turbine engines. While the sensing range is limited by the frequency and the probe can only operate at 900 °C without cooling. As early as 1982, NASA and GE published their cooperative research results of an optical sensor for measuring tip clearance, including test results on the compressor disk [7]. Since 2013, García I. and Zubia J. et al. from University of the Basque Country had been continuously published the results of optical method application in tip clearance measurement [8,9,10,11,12]. While, the study of Andreas K. et.al. [13] proved the optical sensor still have some problems to be solved such as optical fiber heat-resistance, lens cleanness, and the Doppler effect. Capacitive method is the most mature technology so far. Early as 1953, Mossop I.A. et al. [14] published a set of capacitive measurement system for turbine tip clearance. Muller D. et al. [15] conducted the dynamic tip clearance measurement experiment on the compressor and turbine and validated the system uncertainty and stabilization. While the sensor may be influenced by permittivity change of medium and has zero drift problems. Sridhar V. and Chana K.S. et al. [16,17,18,19] used an eddy current probe on the gas turbine engine to obtain tip clearance values in the high pressure turbine stage. The results showed the sensor was able to perform at these extreme environments without losing accuracy. Du L. and Zhu X.L. et al. [20,21] verified the eddy current method in laboratory with 3000 rpm revolution and 1300 K temperature. 

Based on above, eddy current method is a potential way to monitor the dynamic blade tip clearance in turbine. Unfortunately, the measurement signal of inductive sensor is inevitably affected by temperature variation due to its working principle and unavoidable temperature drift problems. Lyu Y.T. et al. [22] used temperature-compensation circuit to eliminate temperature drift of inductive sensor from 20 °C to 500 °C. Wang H.B. et al. [23] reported their experimental finding that resistance has a larger coefficient with temperature change compared to that of inductance, and resistance variation compensates for the influence of temperature on inductance variation. This self-temperature compensation method for ECS is simple and low cost, and has competitive advantages in most applications.

Hence, this paper focused on validating the high-resolution inductive sensor performance and aimed at finding the temperature influence law and using it in the actual turbine measurement at extremely high temperature such as 1000 °C. Furthermore, instead of the complex signal processing circuits [23], a simpler voltage division circuit was used to calculate the coil resistance and coil inductance based on the phasor analysis. The sensor resolution and range were verified by calibration and then the sensor heat-resistance was tested on thermal test bench. Finally, the influence law of temperature on sensor parameters was explored experimentally.

## 2. Method and Sensor 

### 2.1. Inductive Tip Clearance Measurement

**Figure 1:**
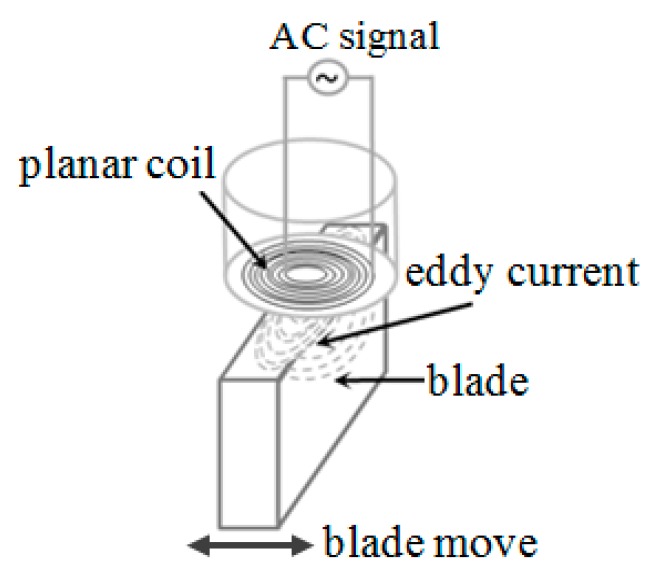
Schematic diagram of measuring principle.

**Figure 2:**
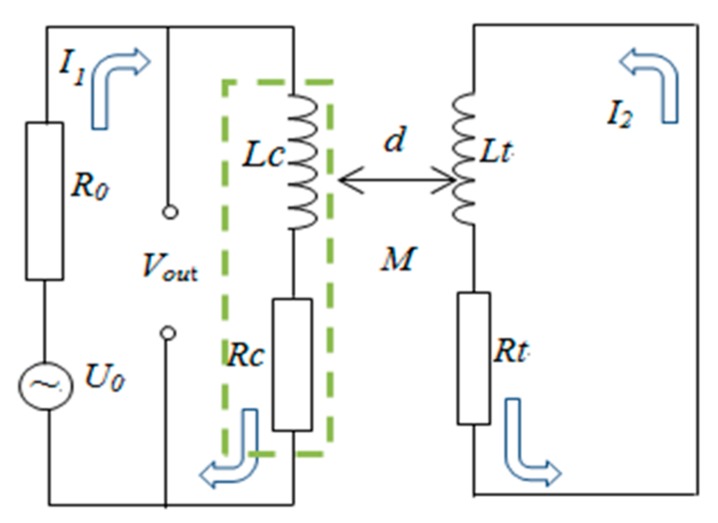
Equivalent circuit of measurement.

The working principle of inductive sensor is based on Faraday’s law of electromagnetic induction and Lenz’s law. The main component of the sensor is an inductive coil, which can be three dimensional spiral coil or two dimensional planar coil. The coil generates a magnetic field when excited by a high frequency AC signal, and then eddy current is induced in the metallic target when it passing through the magnetic field and thus a reverse magnetic flux caused by eddy current declines the inductance of sensor coil. Figure 1 shows the working principle of the inductive sensor (encapsulated planar coil). Figure 2 is the measurement equivalent circuit.

The relation between equivalent circuit parameters and output signal can be derived from the Kirchhoff Voltage Law (Equations (1) snf (2)).

(1) $$R_{c}I_{1}+j\cdot \omega L_{c}I_{1}-j\cdot \omega MI_{2}=V_{out}$$

(2) $$R_{t}I_{2}+j\cdot \omega L_{t}I_{2}-j\cdot \omega MI_{1}=0$$

The equivalent resistance *R*, inductance *L* and impedance *Z* of sensor coil can be derived from Equations (1) and (2), as in Equation (3)–(5):

(3) $$R=R_{c}+\frac{\omega^{2}M^{2}}{R_{t}{}^{2}+{\left(\omega L_{t}\right)}^{2}}R_{t}=R_{c}+R_{e}$$

(4) $$L=L_{c}-\frac{\omega^{2}M^{2}}{R_{t}{}^{2}+{\left(\omega L_{t}\right)}^{2}}L_{t}=L_{c}-L_{e}$$

(5) $$Z=R+j\omega L=\left[R_{c}+R_{e}\right]+j\omega \left[L_{c}-L_{e}\right]$$

Therefore, the equivalent impedance of coil Z is related to the target and the distance between target and sensor coil under the certain excitation signal. The change of equivalent impedance is caused by equivalent resistance *R* and equivalent inductance *L* variation while *R* and *L* are determined by the clearance between coil and target (*M*), exciting voltage frequency (*f*, *f* = 2πω), coil inductance (*Lc*), and coil resistance (*Rc*) as Equations (3) and (4) show. *Rt* is the equivalent resistance of induced eddy current and *Lt* is equivalent inductance of induced eddy current. Under the certain condition, *f*, *Lc* and *Rc* are constants so the equivalent impedance becomes the univalent function of distance (*d*).

### 2.2. Sensor Structure and Manufacture

The magnetic field intensity of circular coil is higher than that of square coil. The magnetic field intensity *B* generated by circular coil is calculated by Equation (6). When the material permeability μ is determined by material property and current *I* is determined by exciting circuit, it can be seen that coil turns (*N*) and coil thickness (*h*) can both influence the magnetic field intensity.

(6) $$B=\mu \frac{N*I}{h}$$

Harold Wheeler [24] had published the research results of eddy current coil in 1928 and Equation (7) is the planar coil inductance formula. It indicates the coil inductance also has positive correlation with coil turns (*N*), radial dimension (*r*), and inverse correlation with thickness (*h*).

(7) $$L\left(\mu H\right)=\frac{r^{2}\times N^{2}}{\left(8r+279.4h\right)}$$

From the above, the magnetic field intensity and coil inductance are proportional to *N* and inversely proportional to *h* when the sensor coil is connected to a certain circuit. That is to say the coil with more turns and thinner thickness can generate higher magnetic field intensity, thus coil has higher sensitivity and wider measuring range.

The study result of Du L. et al. [20] indicated the planar coil had simple geometry, intensive magnetic field and fast response speed, its sensitivity and range satisfied the turbine tip clearance measurement requirement. Thus, the sensor in this paper is also planar coil without iron core, that is to say the minimum *h* equals to the wire diameter and the compact method is adopted to wrap coils to permit more turns for the given coil size and generate higher magnetic flux density.

**Figure 3:**
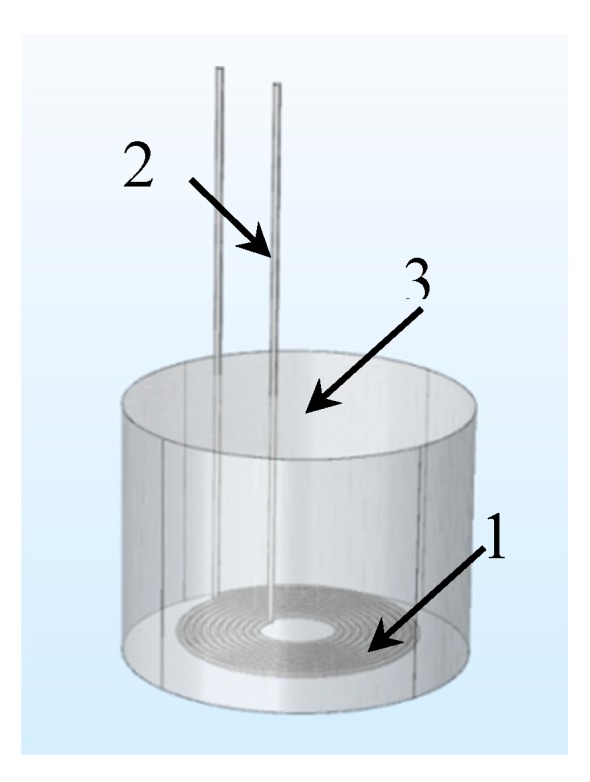
Design sketch of sensor, wherein **1** is leading line, **2** is sealing package, **3** is platinum coil.

**Figure 4:**
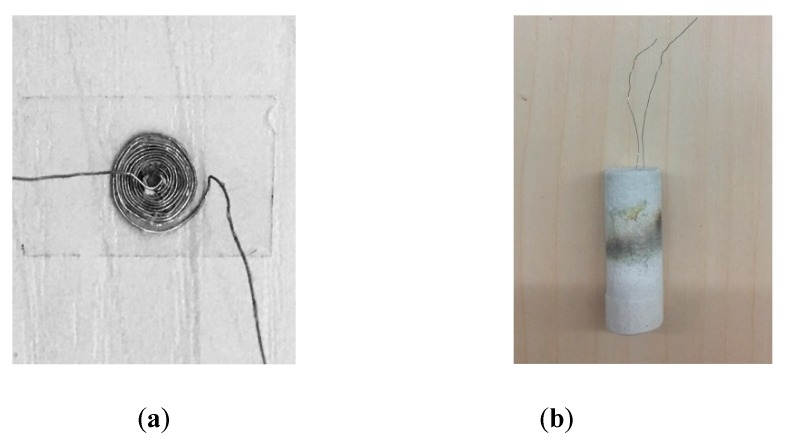
(**a**) Sensor coil before encapsulation; (**b**) sensor after encapsulation.

The sensor probe in this paper was designed as Figure 3 shows, mainly included a planar coil and its sealed ceramic package. According to the study results in [25,26], the planar coil was made of 0.2 mm platinum wire which melting point is over 2000 K. First, drilled 1mm diameter holes in the center of two separate 3 cm × 4 cm acrylic plates and fixed them in parallel and kept the distance slightly over 0.2 mm. Then, a 0.8 mm diameter tube was inserted through the holes and held. The wire was wrapped around the tube between the two plates to form the planar hollow coil. When the planar coil was formed, carefully removed the central tube and the upper plate, and then thin coil was glued off the bottom plate by adhesive tape. Finally, a high temperature ceramic adhesive gel was used to seal the coil to avoid it being corroded at corrosive environment. The sensor used in the paper was an encapsulated 10-turns coil as Figure 4 shows.

In order to evaluate the coil quality, quality factor (*Q*) is used to indicate the quality of inductive elements. The higher *Q* value means the coil is more inductive and effective in the non-contact measuring. The *Q* is calculated by Equation (8):

(8) $$Q=\frac{\omega *L_{c}}{R_{c}}=tan\left(phaseangle\right)$$

where ω is the excitation signal angular frequency and ω = 2∗pi∗*f*, $L_{c}$ is the coil equivalent inductance and $R_{c}$ is the coil equivalent resistance.

The parameters of coil (*Lc*, *Rc*) adopted in the research were measured by LCR meter (*HIOKI IM3536*). The inductance *Lc* and resistance *Rc* are 0.6399 μH and 1.1262 Ω under 4MHz excitation signal. Then the coil *Q* value is calculated as 14.27 and phase angle is 86.2° (the phase angles of inductance elements are usually 45°~90°) which means the coil is a good inductance element.

## 3. Sensor Performance

### 3.1. Characteristics Calibration

**Figure 5:**
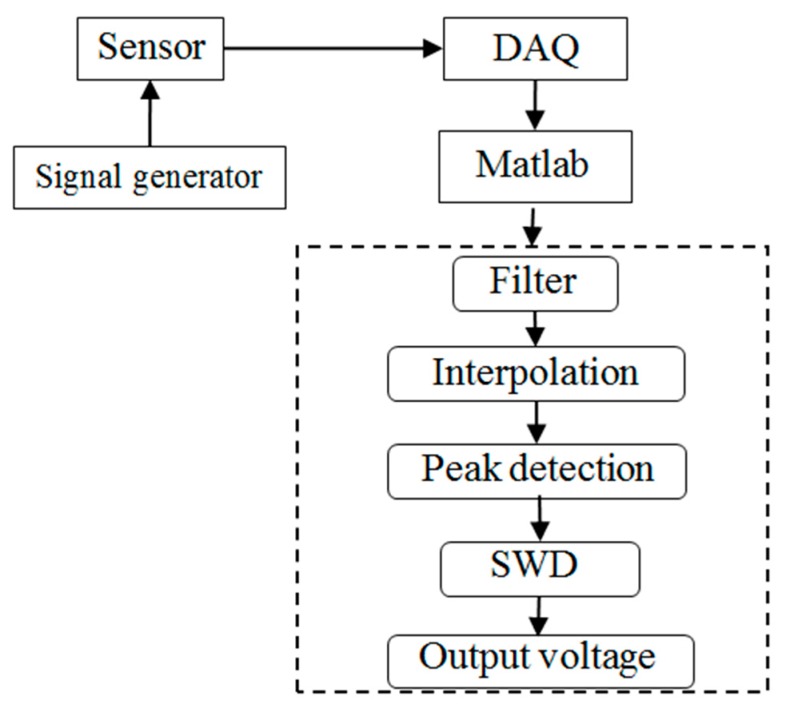
Data processing process. SWD: stationary wavelet decomposition. DAQ: data acquisition.

**Figure 6:**
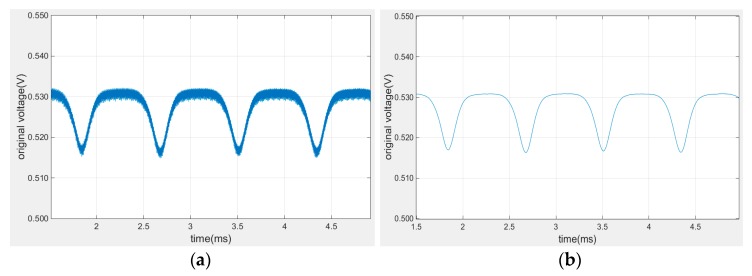
SWD effect comparison; (**a**) signal sequence before SWD; (**b**) signal sequence after SWD.

In order to verify the sensitivity and measuring range of designed platinum coil, the calibration experiment was conducted at room temperature to obtain the sensor characteristic curve. The characteristic curve of the tip clearance sensor refers to the relationship curve of sensor characteristic parameter and the clearance. The relative variation of voltage was used as measuring quantity in the study. Data processing process is shown in Figure 5. The signal was collected by DAQ (Data Acquisition) and then input into Matlab. In order to increase the signal-to-noise ratio (SNR), FFT method was used to remove the high frequency noise and stationary wavelet decomposition (SWD) method was used to smooth the voltage signal. Figure 6 is the comparison of the signal sequence in time domain before and after SWD. It is proved that SWD can efficiently suppress high frequency noise and increase the SNR of voltage signal.

The calibration system includes sensor probe, calibration target, position controller, function generator, and DAQ system. The calibration target is a 10 mm width and 1.5 mm thickness plate, and the target material is Inconel 718 which is one of the turbine blade materials. The precision of position controller is 1 μm and its control range is 13 mm. The excitation signal in the calibration experiment was 4 MHz and 3 Vpp sinusoidal AC signal generated by *Agilent Keysight 33600A (Agilent Technologies Inc., Santa Clara, CA, USA)*.

After connecting the measuring circuit (function generator, sensor probe and DAQ card) and fixing the target position, collected the voltage V_0_ on the sensor coil. Then, adjusted the distance between sensor surface and target surface from 0mm to 5 mm with 50 μm step, and collected voltage signals on the sensor coil at each position. Voltage variation dV was denoted as sensor characteristic parameter and calculated in Matlab.

**Figure 7:**
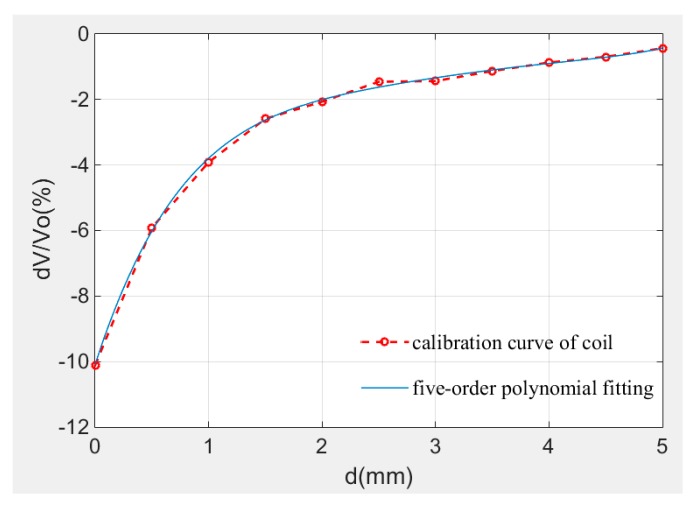
Calibration curve at room temperature.

The calibration result is shown in Figure 7. Equation (9) is five order fitting formula and its fitting degree R² is 0.9992. The signal data near 5mm is listed in Table 1.

(9) $$\mathrm{dV}/V_{0}=a_{5}\times d^{5}+a_{4}\times d^{4}+a_{3}\times d^{3}+a_{2}\times d^{2}+a_{1}\times d+a_{0}$$

wherein, a_0_ = 10.1, a_1_ = 10.7, a_2_ = −5.86, a_3_ = 1.73, a_4_ = −0.259, a_5_ = 0.0155.

According to the calibration curve in Figure 7, the sensor has good sensitivity (4%/mm) within 2 mm. The sensitivity and the resolution of the sensor decrease with the distance as Figure 7 shows and the data precision of measurement system is 0.001%, data listed in Table 1 proves the measuring range of the sensor is over 5 mm and the resolution reaches 10 μm for the data still has 0.07% variation at 5 mm.

**Figure 8:**
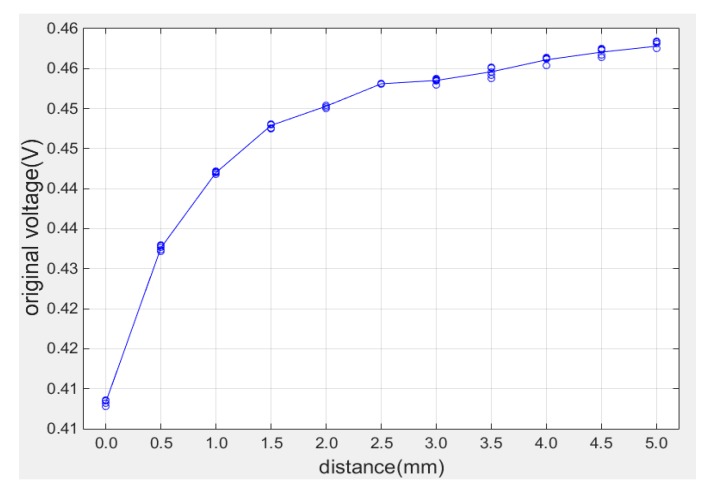
Repeatability measurement at different position.

In order to verify the accuracy of calibration results, the repeatability of measuring signal of 11 position points within 0–5 mm range was calculated with 95.56% confidence interval. Ten sets of data were collected at each position point and the measurement results in the whole range are plotted in Figure 8. The relative standard deviation can be calculated by Equation (10):

(10) $$RSD=\frac{2*S_{X}}{\bar{X}}\times 100\%$$

$where\;S_{X}$ is standard deviation based on the sample population, $\bar{X}$ is arithmetic mean of the sample.

From the data in Table 2, the measuring repeatability at different positions is almost within 0.05% which means the coil had good repeatability within measuring range.

### 3.2. Heat Resistance Test

**Figure 9:**
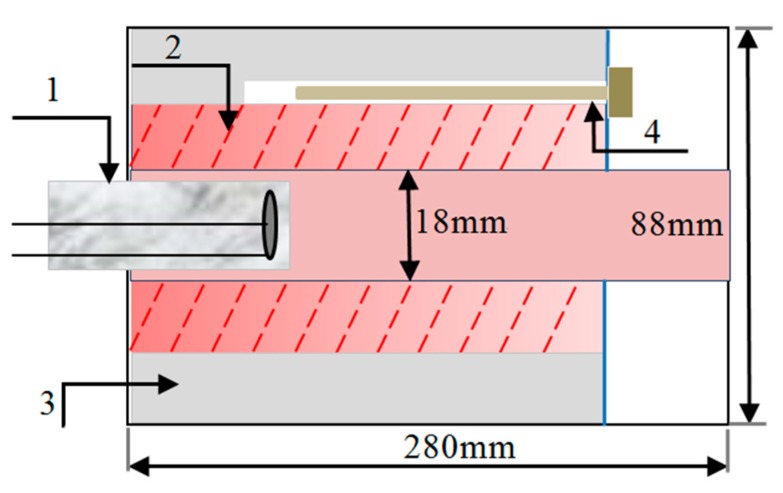
Static thermal test rig scheme (1—sensor, 2—heating rod, 3—insulating layer, 4—thermocouple).

**Figure 10:**
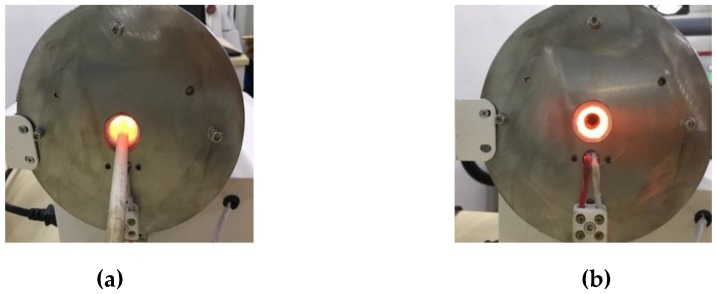
Test rig at 1100 °C; (**a**) sensor position; (**b**) inside the heater.

The purpose of heat resistance test is validating the sensor can withstand the actual operating environment temperature over 1000 °C. The heat resistance of the sensor was verified according to the stability and repeatability of the output signal during the multiple thermal cycles. The thermal cycling test was conducted on the static test rig as Figure 9 shows, it is actually a tubular heater with an accurate temperature controller, which control precision is ±1 °C Figure 10 shows the test rig at 1100 °C.

**Figure 11:**
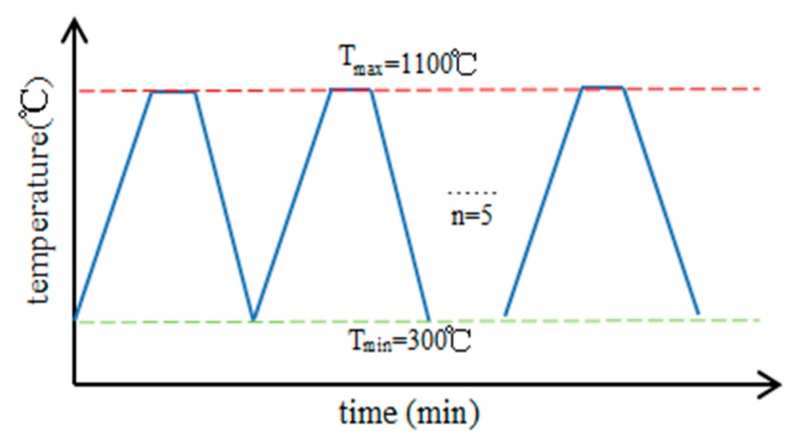
Temperature cycling curve.

In the process of thermal cycle, temperature increased from 300 °C to 1100 °C (~1373K) after 60 min and maintained at 1100 °C for 2 h, then cooled down to 300 °C after 40 min, and repeated this cycle for five times as the operating condition curve shown in Figure 11. 

**Figure 12:**
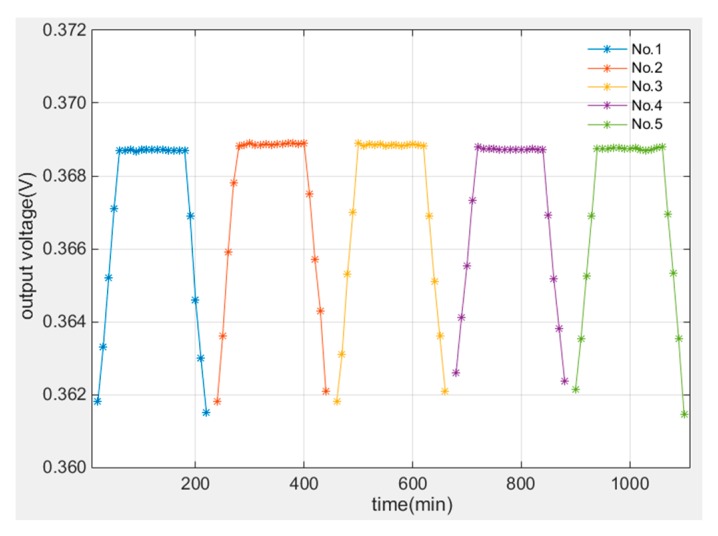
Heat resistance test results at high temperature.

The measuring result of voltage on sensor is demonstrated in Figure 12. The result indicates the sensor voltage remained stable in high temperature duration which proves the sensor can keep reliable at least 2 h in 1100 °C environment and the sensor voltage changed with temperature similarly during the heating and cooling processes.

## 4. Thermal Effect on Sensor

Based on the principle of eddy current sensing, temperature change will leads to the coil parameters like permeability and conductivity change. Then the measuring results will be influenced. In order to know how the inductive sensor can be affected by high temperature, experiment method was used to get the influence of temperature on sensor.

### 4.1. Experiment Method

**Figure 13:**
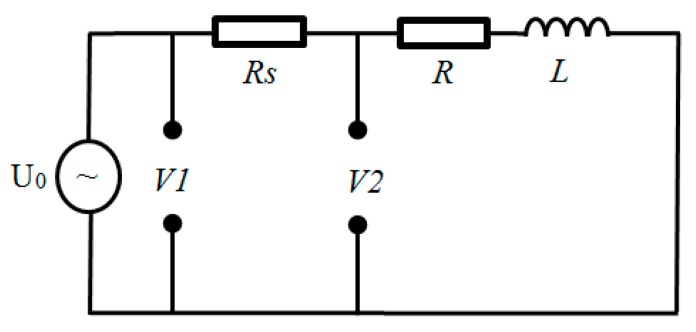
Equivalent measurement circuit of measure coil parameters (*L*, *R*).

The research adopted the equipment as Figure 9 and Figure 10 show to create the adjustable temperature environment and measure coil parameters (*L*, *R*) without target under test at different temperature. The experiment temperature increased from 500 °C to 1100 °C. The equivalent measurement circuit of LR is shown as Figure 13. The sensor coil is equivalent to a series resistor R and a pure inductor L. The divider resistor *Rs* was 7.5 Ω and the excitation signal was 4 MHz 3 Vpp AC voltage (U_0_). When the environment temperature changes, coil inductance *L* and coil resistance *R* may both vary. 

**Figure 14:**
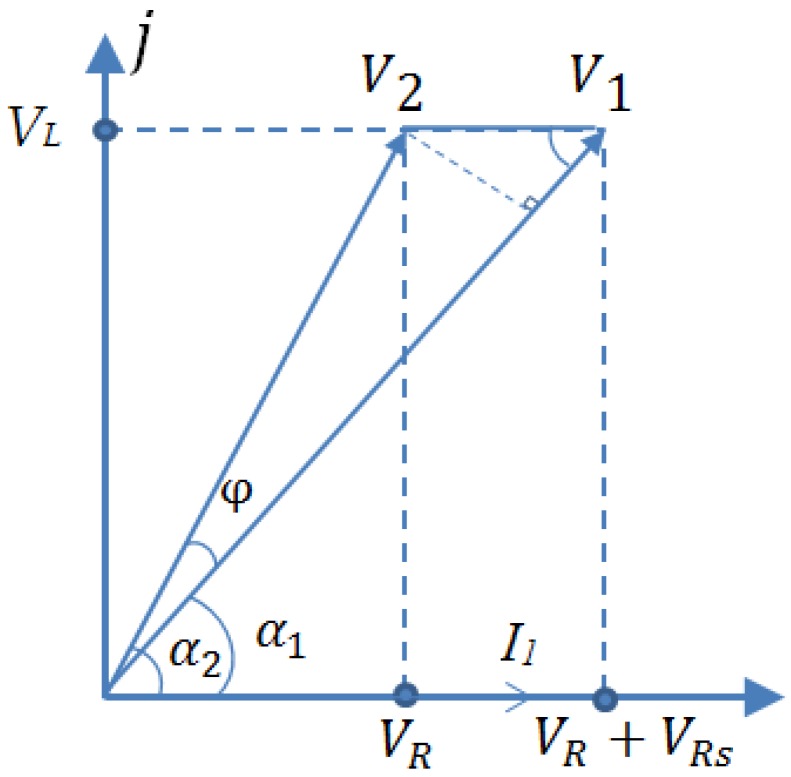
Phasor representation of measured signal.

The measurement circuit parameters are represented by phasor as Figure 14 shows. *V1* is divide voltage on the *Rs* and coil while *V2* is divide voltage on the coil. According to Figure 13 and Figure 14, formulas for calculating sensor resistance and inductance were derived as Equation (11)–(15) show. The measured original data (*V1*, *V2*) was processed in MATLAB to be filtered through FFT method and then coil inductance *L* and resistance *R* were calculated under different temperature. Then the variation law of coil parameters with temperature is obtained.

(11) $$I_{1}=\frac{V1-V2}{R_{s}},$$

(12) $$\alpha_{1}=arcsin\frac{V2\cdot sin\varphi }{I_{1}R_{s}},$$

(13) $$\alpha_{2}=\alpha_{1}+\varphi ,$$

(14) $$R=\frac{\left|V2\right|\cdot cos\alpha_{2}}{\left|I_{1}\right|},$$

(15) $$L=\frac{\left|V2\right|\cdot sin\alpha_{2}}{\omega \cdot \left|I_{1}\right|}$$

where $I_{1}$ is the current in the LR circuit, φ is the phase difference between *V1* and *V2*, $\alpha_{1}$ and $\alpha_{2}$ are the phase angle of *V1* and *V2* respectively.

### 4.2. Experiment Results

**Figure 15:**
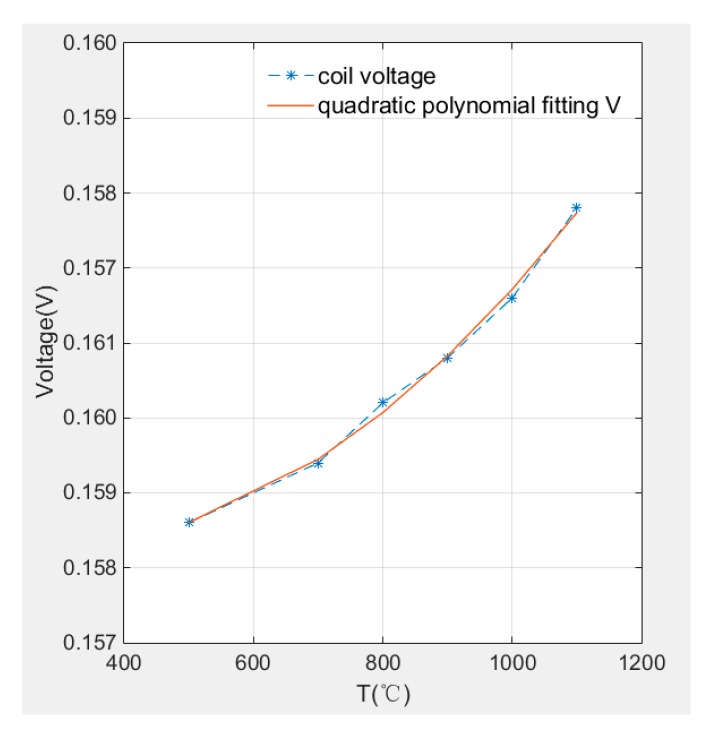
Coil voltage curve with temperature.

The relationship of sensor voltage and temperature is plotted in Figure 15. It can be observed that the sensor voltage and temperature has quadratic relationship so the voltage can be expressed by quadratic polynomial fitting expression as Equation (16) shown and the R^2^ equals to 0.9999, under the condition that the temperature range is 500 °C to 1100 °C.

V(T) = 0.15791 − 1.8905 × 10^−6^ × T + 3.3333 × 10^−9^ × T^2^ (16)

**Figure 16:**
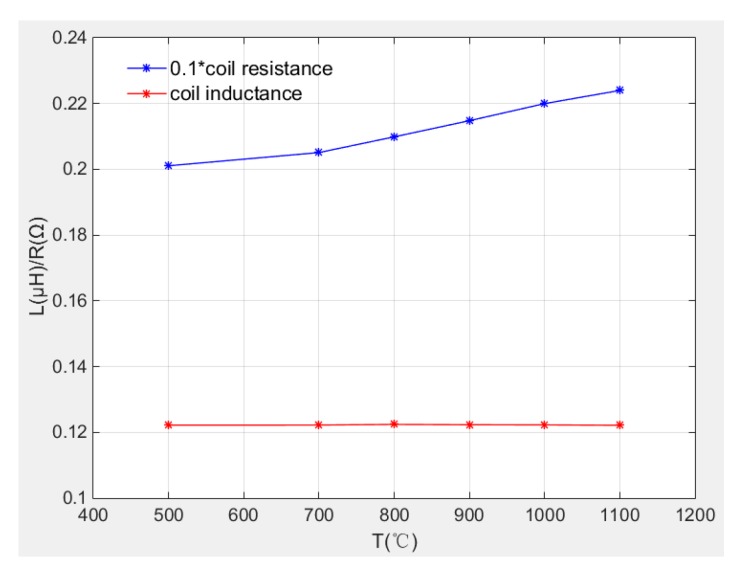
Comparison of L and R under different temperature.

The coil resistance and inductance at different temperatures are compared in Figure 16. It is clearly that the coil resistance increases with temperature especially between 700 °C to 1100 °C, the resistance increment is up to 11.4% from 500 °C to 1100 °C.

The coil inductance is almost unchanged when temperature increased from 500 °C to 1100 °C. The inductance remained stably about 0.1223 μH and the maximum inductance fluctuation is 0.343%.

**Figure 17:**
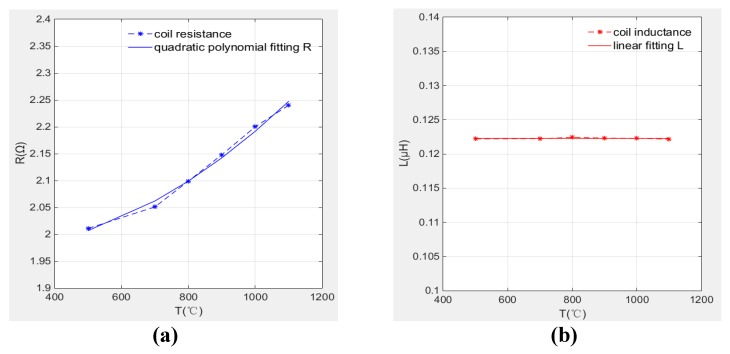
Coil parameters change curve with temperature; (**a**) resistance; (**b**) inductance.

Figure 17a,b illustrate the variation of resistance and inductance with temperature respectively. It is also clearly that the resistance has quadratic relationship with temperature. This relationship can be used to indicate operating temperature without need for additional thermocouple. Therefore, in the application of distance measurement, coil inductance is determined as measurement parameter to avoid the temperature drift problems. 

Meanwhile, compared with linear fitting and higher order fitting, the fitting degree of quadratic fitting is good enough and in order to facilitate subsequent back-extrapolation of ambient temperature through resistance values, the coil resistance and inductance are expressed by quadratic fitting relation and linear fitting relation as Equations (17) and (18) show. The polynomial coefficients are listed in Table 3.

R(T) = p_0_ + p_1_ ∗ T + p_2_ ∗ T^2^   R^2^ = 0.9829 (17)

L(T) = p_0_ + p_1_ ∗ T    R^2^ = 0.9998 (18)

Based on the above discussions, it is found by experiment that temperature has obvious influence on induction coil resistance while the coil inductance almost does not change with temperature. Hence, in the tip clearance measurement, the coil inductance value is determined as measurement variable for it is not affected by temperature changes, that is to say the characteristic curve of the sensor becomes the relationship curve between coil inductance and the clearance. While, the resistance value change can indicate the environment temperature change. In this way, an inductive sensor can measure tip clearance as well as measure the environment temperature through the data processing.

## 5. Conclusions

Based on Faraday’s law of electromagnetic induction and Lenz’s law, the following conclusions are obtained by experiments:

1. The designed sensor with planar coil made of platinum wire is proved to be a good inductive sensor for its phase angle is up to 85° and quality factor is 14.27 under 4 MHz excitation frequency.
2. The sensor performance meets the requirements of tip clearance measurement for the measuring range of sensor is proved to be at least 5 mm and the resolution is better than 10 μm within 5 mm range according to static calibration result. It is also found that the sensor coil repeatability is almost better than 0.05% within the whole sensing range.
3. The encapsulated platinum coil can be long-term (2 h) heat-resistant at 1100 °C and maintains a good stability during multiple temperature cycles. This suggests the designed sensor is capable to operate in the high temperature for a long time and that is an important basis for the sensor to be used in turbine tip clearance measurement in the future.
4. The inductance and resistance of the sensor coil can be solved based by phasor analysis and using the series resistance circuit. This decoupled analysis of sensor parameters makes its application range wider.

## Figures and Tables

**Table 1:** Calibration data at near full range area.

Clearance (mm)	dV/V_0_ (−%)
4.95	0.465
4.96	0.457
4.97	0.450
4.98	0.442
4.99	0.434
5.00	0.427

**Table 2:** Repeatability measurement data.

Clearance (mm)	Voltage (V)	Repeatibility (%)
0.0	0.41332	0.0327
0.5	0.43260	0.0300
1.0	0.44202	0.0171
1.5	0.44786	0.0288
2.0	0.45032	0.0179
2.5	0.45314	0.0055
3.0	0.45352	0.0311
3.5	0.45458	0.0593
4.0	0.45614	0.0483
4.5	0.45706	0.0378
5.0	0.45781	0.0327

**Table 3:** Polynomial coefficient of R and L.

	p_0_	p_1_	p_2_
R(T)	1.9779	−9.7757 × 10^−5^	3.1143 × 10^−7^
L(T)	0.12227	−2.8571 × 10^−9^	/
